# TLR2 and AP-1/NF-kappaB are involved in the regulation of MMP-9 elicited by heat killed *Listeria monocytogenes* in human monocytic THP-1 cells

**DOI:** 10.1186/s12950-015-0077-0

**Published:** 2015-04-18

**Authors:** Puthiyaveetil Kochumon Shihab, Areej Al-Roub, Moneera Al-Ghanim, Anfal Al-Mass, Kazem Behbehani, Rasheed Ahmad

**Affiliations:** Immunology & Innovative Cell therapy Unit, Dasman Diabetes Institute, Al-Soor Street, P.O. Box 1180, Dasman, 15462 Kuwait

## Abstract

**Background:**

MMP-9 is crucial for a normal immune response, but excessive release of this enzyme leads to severe tissue damage. *Listeria monocytogenes* (LM) is an opportunistic food-borne pathogen causing listerosis, meningitis and sepsis. Heat killed *Listeria monocytogenes* (HKLM) activates immune system and leads production of cytokines and chemokines. However, nothing is known about the involvement of HKLM in MMP-9 regulation. Therefore we investigated the role of HKLM in the regulation of MMP-9 gene expression in THP-1 cells.

**Methods:**

Commercially available heat killed *Listeria monocytogenes* was used in this study. HKLM-induced MMP-9 expression was assessed with quantitative real-time qPCR and ELISA. Action of HKLM in different signaling pathways were studied by using THP-1-XBlue™ cells (THP-1-cells with NF-κB/AP-1 reporter construct), THP-1-XBlue™-defMyD cells (MyD88^−/−^ THP-1 cells), anti-TLR2 mAb and pharmacological inhibitors. Phospho and total proteins were determined by Western blotting.

**Results:**

Increased MMP-9 production (mRNA: 395-Fold; Protein: 8141 pg/ml; P < 0.05) was observed in HKLM stimulated THP-1 cells as compared to the un-stimulated THP-1 cells. This production of MMP-9 was completely abrogated by anti-TLR2 blocking mAb (P = 0.0024). Furthermore, THP-1-XBlue™-defMyD cells were unable to produce MMP-9 in response to HKLM. HKLM- induced activation of NF-kappaB/AP-1 was also observed in THP-1-XBlue™ Cells. In addition, inhibitors of JNK (SP600125), MEK/ERK (U0126; PD98056), p38 MAPK (SB203580) and NF-kappaB (BAY 11–7085, Triptolide and Resveratrol) significantly suppressed (*P* < 0.05) HKLM-stimulated MMP-9 expression.

**Conclusion:**

Our results indicate that HKLM activates TLR2 and NF-κB/AP-1 signaling pathways, leading to up-regulation of MMP-9 production in THP-1 cells. Thus, MMP-9 could be an appropriate therapeutic target to stop severe tissue damage caused by infection or chronic inflammation.

## Introduction

*Listeria monocytogenes* is a Gram-positive foodborne pathogen that is widely distributed in nature, occurring in soil, water, various food products, animals, and humans [1]. Infection by Listeria monocytogenes occurs almost exclusively after ingestion of contaminated food [2]. Immunocompromised individuals, neonates, pregnant woman, elderly persons, and patients suffering from transplantation events are most susceptible to infections. Listeriosis causes invasive disease including septicemia and meningitis [3]. Although the listeriosis incidence is low, the high mortality rates (about 24%) due to septicemia and meningitis make L. monocytogenes one of the most deadly human food-borne pathogens [4]. Immediate immune responses are triggered during LM infection. Innate immunity to LM is mediated via toll like receptors or nucleotide-binding oligomerization domain (NOD)-like receptors (NLRs) [5]. Toll-like receptors (TLRs) have been shown to play an important role in the host’s innate immune responses to microbial infections through the induction of proinflammatory cytokines, chemokines, and type I interferons by macrophages and dendritic cells [6,7]. Member of the TLR family, namely TLR2 has been shown to be critical in the initiation of innate immune responses to LM infection in the mouse model [8,9]. Recognition of a microbial invasion through the TLRs triggers the activation of signaling pathways, resulting in the recruitment of several adaptor proteins to the TIR domain. However, myeloid differentiation factor 88 (MyD88) is a key adaptor protein which is common to almost all TLRs except TLR3 [10]. MyD88 activates in turn IL-1 receptor–associated kinases (IRAK) family members and tumor necrosis factor-alpha receptor–associated factor 6 (TRAF6) [11,12]. These adaptor proteins have essential role in the activation of NF-κB and mitogen-activated protein kinase (MAPK) pathways [13-18]. NF-kappaB and AP-1 transcription pathways are involved in the regulation of inflammatory mediators that trigger the migration of the inflammatory cells into the tissue. Inflammatory cells migration into tissues is dependent on several events including adherence to endothelial cells and penetration through the vessel wall into the extracellular matrix [19-21].

Matrix metalloproteinases (MMPs) form a family of zinc-containing proteases that degrade all extracellular matrix components and have an important role in tissue remodeling and immunomodulatory functions [22,23]. As gelatin is a major component of extra- cellular matrix (ECM) and in view of their collagen type IV-specific degradation capacity, MMP-9 plays a key role in ECM breakdown. MMP-9 is predominantly secreted by monocytes which are central cells in developing immune response to infection. The production of MMP-9 by monocytes is of interest in the context of facilitating leukocyte infiltration into infected sites through degrading type IV collagen in vascular basement membranes [24]. MMP-9 production is tightly controlled at the level of gene transcription and its unrestricted release/activity may contribute to host tissue damage during infection. Elevated levels of MMP-9 were found in different inflammatory and infectious diseases [25-28]. MMP-9 gene expression in monocytic cells is regulated by different cytokines, including TNF-alpha IL-1beta, IL-18 and microbial components [29-31].

Previous work has shown that Heat killed listeria monocytogenes (HKLM) activates immune system by regulating the expression of cytokines (IL-1β, IL-6, IL-8, IL-12 and TNFα and chemokines [32-34]. However, nothing is known about the regulation of MMP-9 by HKLM in monocytic cells. In this study we therefore looked at the influence of HKLM on monocyte production of MMP-9. We show that HKLM induces MMP-9 in the monocytic cell line THP-1 via activation of MAPK and NF-kappaB. MMP-9 secretion was blocked by neutralizing TLR2. MyD88−/− cells abrogate the HKLM stimulated MMP-9 secretion.

## Materials and methods

### Cell culture and stimulation

Human monocytic leukemia cell line THP-1 was purchased from American Type Culture Collection (ATCC) and grown in RPMI-1640 culture medium (Gibco, Life Technologies, Grand Island, USA) supplemented with 10% fetal bovine serum (Gibco, Life Technologies, Grand Island, NY, USA), 2 mM glutamine (Gibco, Invitrogen, Grand Island, NY, USA), 1 mM sodium pyruvate, 10 mM HEPES, 100ug/ml Normocin 50 U/ml penicillin and 50 μg/ml streptomycin (P/S; (Gibco, Invitrogen, Grand Island, NY, USA), and incubation at 37°C (with humidity) in 5% CO_2_. THP-1-XBlue cells stably expressing a secreted embryonic alkaline phosphatase (SEAP) reporter inducible by NF-κB and AP-1 were purchased from InvivoGen (InvivoGen, San Diego, CA, USA). THP-1-XBlue cells show similar response to HKLM purchased from InvivoGen, (San Diego, CA, USA) as THP-1 cells. All the experiments were performed with THP-1-XBlue cells at a cell density of 1 × 10^6^ /ml in 12-well plates. THP-1-XBlue™-defMyD cells (cells deficient in MyD88 activity; MyD88−/− THP-1 cells) were also purchased from InvivoGen (InvivoGen, San Diego, CA, USA). THP-1-XBlue cells were cultured in complete RPMI medium with the addition of zeocin (200 μg/ml) (InvivoGen, San Diego, CA, USA) to select for cells expressing the SEAP -NF-κB/AP-1 reporter. THP-1-XBlue™-defMyD cells were cultured in complete RPMI medium with the addition of Zeocin (200ug/ml) and HygroGold (100ug/ml) (InvivoGen, San Diego, CA, USA).

Prior to stimulation, THP-1 cells were transferred into normal medium and plated in 12-well plates (Costar, Corning Incorporated, Corning, NY, USA) at 1 × 10^6^ cells/well cell density unless indicated otherwise. In dose–response experiment, the following HKLM concentrations were used to stimulate THP-1 cells: 1-9×10^7^ particles/ml. In subsequent experiments, the optimal (non-cytotoxic) concentration of HKLM (3×10^7^ particles/ml) or TNF-alpha (25 ng/ml) were used to stimulate cells for 24 hr at 37°C. Cells were harvested for RNA isolation and conditioned media were collected for measuring MMP-9 secretion levels and SEAP activity. Conditioned media were collected and stored at −80°C.

### Quantification of NF-κB/AP-1 activity

THP-1 XBlue cells (InvivoGen, San Diego, CA) are THP-1 cells stably transfected with a reporter construct, expressing a secreted embryonic alkaline phosphatase (SEAP) gene under the control of a promoter inducible by the transcription factors NF-κB and AP-1. Upon stimulation, NF-κB and AP-1 are activated and subsequently the secretion of SEAP is promoted. Levels of SEAP were detected in the conditioned media after 4 hr incubation of supernatants with Quanti-Blue medium (InvivoGen, San Diego, CA, USA) at 650 nm wave length by ELISA reader.

### Real time quantitative RT-PCR

Total RNA was extracted using RNeasy Mini Kit (Qiagen, Valencia. CA, USA). The cDNA was synthesized using 1 μg of total RNA using high capacity cDNA reverse transcription kit (Applied Biosystems, Foster city, CA, USA). 50 ng cDNA was used in each real-time PCR reaction. For real-time polymerase chain reaction (PCR), complementary DNA was amplified with Inventoried TaqMan Gene Expression Assay products (MMP-9: Hs00234579_m1; GAPDH: Hs03929097_g1) containing two gene-specific primers and one TaqMan MGB probe (6-FAM dye-labeled) using a TaqMan® Gene Expression Master Mix (Applied Biosystems, Foster city, CA, USA) in a 7500 Fast Real-Time PCR System (Applied Biosystems, Foster City, CA, USA). The mRNA levels were normalized against GAPDH mRNA and the amounts of MMP-9 mRNA relative to control were calculated with ΔΔCt-method [35]. Relative mRNA expression was expressed as fold expression over average of control gene expression. The expression level in control treatment was assumed to be 1. Values are presented as mean ± SEM. Results were analyzed statistically; *P* < 0.05 was considered significant.

### ELISA for secreted MMP-9 and TIMP-1 in cell culture supernatants

Concentrations of MMP-9 and TIMP-1 in cell culture supernatants were measured using sandwich ELISA according to the manufacturer’s instructions (R&D systems, Minneapolis, USA).

### Western blotting

Cellular lysates were prepared as described previously [7,36]. Briefly THP-1 cells were incubated for 30 min with lysis buffer (Tris 62.5 mM (pH 7.5), 1% Triton X-100, 10% glycerol). The lysates were then centrifuged at 14000 rpm for 10 min and the supernatants were collected. Protein concentration in the lysates was measured by Quickstart Bradford Dye Reagent, 1x Protein Assay kit (BioRad Laboratories, Inc, CA). Protein (20 μg) samples were mixed with sample loading buffer, heated for 5 min at 95°C and were resolved on SDS-12% SDS-PAGE and transferred to immobilon polyvinyldifluoride (PVDF) membranes (Bio-Rad Laboratories, USA) by electro blotting. The blots were blocked with 5% non-fat milk in PBS at room temperature and then probed with rabbit anti-human antibodies against p-MEK1/2, pERK1/2, p-JNK, p-p38, p-c-jun, p-IKKα/β, p-IKB, p-NF-kappaB and Beta Actin in 1:1000 dilution at 4°C overnight. All the primary antibodies were purchased from Cell Signalling (Cell Signalling Technology, Inc). The blots were then washed three times with TBS and incubated for 2 h with HRP-conjugated secondary antibody (Promega, Madison, WI, USA). Immunoreactive bands were developed using an Amersham ECL Plus Western Blotting Detection System (GE Health Care, Buckinghamshire, UK) and visualized by Molecular Imager ® *VersaDoc*™ MP Imaging Systems (Bio-Rad Laboratories, Hercules, CA, USA).

### Statistical analysis

Statsitical analysis was performed using GraphPad Prism software (La Jolla, CA, USA). Data are shown as mean ± standard deviation values, unless otherwise indicated. Unpaired Student t-test was used to compare means between groups. In all cases, *P* value < 0.05 was considered significant.

## Results

### HKLM induces MMP-9 production in THP-1 cells

**Figure 1:**
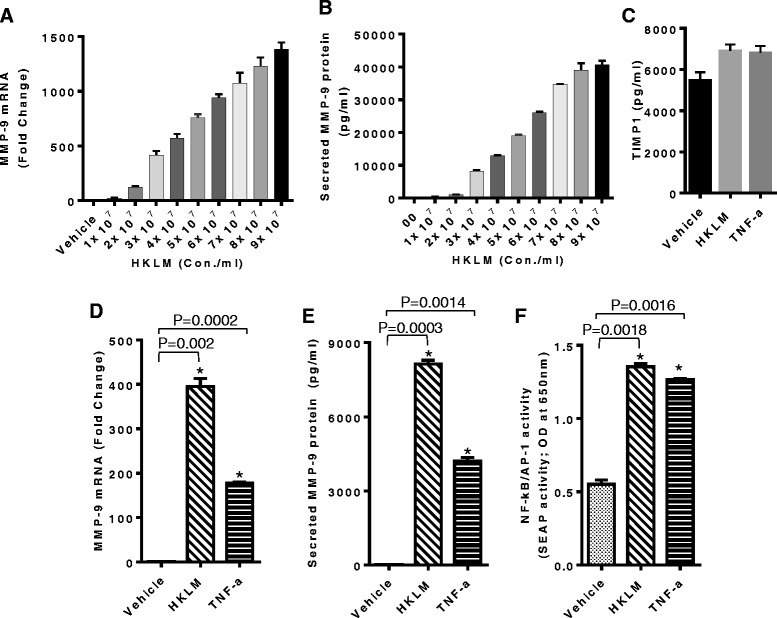
HKLM up-regulates MMP-9 expression in THP-1 cells. THP-1 cells were stimulated with HKLM different concentrations (1-9x10^7^ particles/ml). Cells and culture supernatants were collected. Total cellular RNA was isolated and MMP-9 mRNA was quantified by real time PCR. Relative mRNA expression was expressed as fold expression over average of gene expression in vehicle-treated cells. The average gene expression level in vehicle-treated cells was assumed to be 1 **(A)**. Secreted MMP-9 levels were measured in supernatants by ELISA **(B)**. THP-1 cells were stimulated with HKLM (3x10^7^/ml), TNF-alphalph (25 ng/ml; positive control) and vehicle (H_2_O; 2ul/ml) for 24 hrs. Cells and culture supernatants were collected. MMP-9 mRNA was quantified by real-time PCR **(D)** and secreted levels of TIMP-1 and MMP-9 were measured in the supernatants by ELISA **(C & E)**. SEAP reporter activity (degree of NF-κB /AP-1 activation) was determined in supernatants as described in materials and methods **(F)**. The results obtained from three independent experiments are shown. The data are presented as mean ± SE. An asterisk (*) represents *P*-value of <0.05.

It has been reported that heat killed listeria monocytogenes (HKLM) triggers the production of inflammatory mediators [32-34,37]. However, MMP-9 induction by HKLM has yet not been studied in monocytic cells. To determine whether HKLM up-regulates MMP-9 gene expression, THP-1 cells were treated with different concentrations of HKLM for 24 hrs. Cells and conditioned media were harvested. MMP-9 gene and protein expression were induced in THP-1 cells in a dose-dependent manner (Figure 1A & B, respectively). We further found that HKLM concentration of 3x10^7^ particles/ml did not induce changes in cellular morphology and viability; therefore, this concentration was used in all subsequent experiments. In addition, we also confirmed that the production of TIMP-1 protein differed non-significantly between vehicle and HKLM (Figure 1C). MMP-9 gene expression was increased at both mRNA (395 Fold; P = 0.002) and protein levels (8141 ± 215 pg/ml; P = 0.003) in THP-1 cells treated with HKLM as compared to the un-stimulated THP-1 cells. MMP-9 protein expression induced by TNF-alpha (4195 ± 157 pg/ml; P = 0.0014) as positive control (Figure 1D & E).

Studies have revealed an essential role of NF-kappaB and AP-1 activation in MMP-9 secretion in different cell types by several external stimuli [38,39]. To investigate the involvement of NF-kappaB and AP-1 in HKLM induced MMP-9 gene expression, the THP-1 X-blue cells, expressing a reporter gene SEAP driven by NF-kappaB and AP-1 response elements, were treated with HKLM or TNF-alpha. Elevated SEAP activity (NF-kappaB/AP-1 activation; P = 0.0018) was determined in the condition media obtained from THP-1 cells treated with HKLM as compared to unstimulated cells (Figure 1F).

### Involvement of TLR2 in HKLM induced MMP-9 production

**Figure 2:**
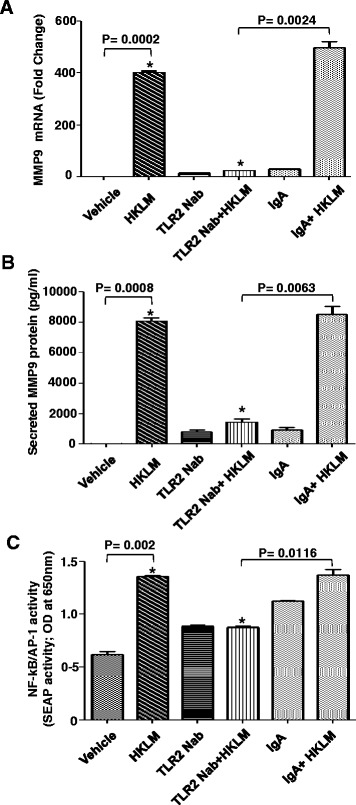
Effect of TLR-2 neutralizing antibody on the induction of MMP-9 expression by HKLM. THP-1 cells were treated anti-huTLR-2-IgA (neutralizing mAb; Nab; 2ug/ml) or isotype-matched control antibody (IgA) (2ug/ml) for 30 minutes before the addition of HKLM. Cells and cell culture supernatants were collected at 24 hr after HKLM treatment. MMP-9 mRNA was quantified by real-time PCR **(A)** and secreted levels of MMP-9 were measured in the supernatants by ELISA **(B)**. SEAP reporter activity (degree of AP-1/NF-κB activation) was determined in the supernatants as described in the material methods **(C)**. Data are shown from three independent experiments. Data are presented as mean ± SE. An asterisk (*) represents *P*-value of <0.05.

Previous studies have shown that TLR2 can interact with several ligands including listeria monocytogenes [40]. Therefore, we hypothesized that the TLR2 signaling pathway might be involved in HKLM-induced MMP-9 production. To test this hypothesis, THP-1 cells were treated with anti-TLR2-neutralizing monoclonal antibody (a-TLR2 mAb) or a control isotype (IgA) for 30 min. The cells were then treated with HKLM and evaluated for MMP-9 gene expression. Neutralization of TLR2, with an anti-TLR2 mAb markedly reduced HKLM–induced MMP-9 gene up-regulation in THP-1 cells (Figure 2A and B; P < 0.05). In this condition, MMP-9 gene expression levels were similar to those seen in non-treated cells, whereas there was no change in MMP-9 expression in cells treated with the control isotype antibody. Furthermore, consistent with the observed effect of neutralization of TLR2 on the induction of MMP-9, HKLM -induced NF-κB/AP-1 activity was significantly reduced (P < 0.05) in THP-1 cells pretreated with anti TLR2 neutralizing antibody compared with control antibody (Figure 2C). These results reveal the absolute requirement of TLR2-mediated signaling for MMP-9 gene up-regulation by HKLM in THP-1 cells.

### Role of MyD88 in HKLM induced MMP-9 production

**Figure 3:**
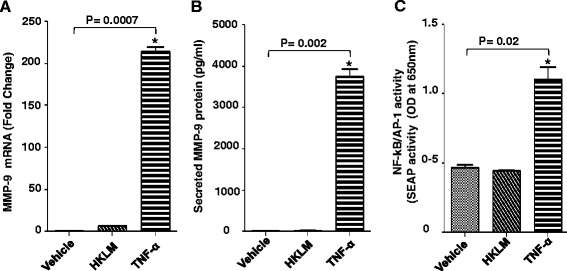
Effect of MyD88 deficiency on the induction of MMP-9 by HKLM. THP-1-XBlue™-defMyD cells (Cells deficient in MyD88 activity) were treated with HKLM (3x10^7^/ml), TNF-alpha (25 ng/ml) and vehicle (water; 2ul) for 24 hr. Cells and supernatants were collected. Cells were used for the isolation of total RNA and MMP-9 gene expression was quantified by real-time PCR **(A)**. Secreted levels of MMP-9 protein were determined in supernatants by ELISA **(B)**. SEAP reporter activity (degree of AP-1/NF-κB activation) was also determined in cell supernatants **(C)**. Data are shown as mean ± SE. An asterisk (*) represents *P*-value of <0.05.

We found in earlier experiments that TLR2 is required for HKLM-mediated induction of MMP-9 production in THP-1 cells and that only HKLM interaction with cellular receptor on the target cells was responsible for inducing this effect. MyD88 appears to be a key adaptor protein as it is required for signalling by all TLRs except TLR3 [10]. To investigate the role of MyD88 in regulation of MMP-9 by HKLM, THP-1-XBlue™-defMyD cells (MyD88−/−; cells deficient in MyD-88 activity) were incubated with HKLM or TNF-alpha. MyD88 deficiency diminished HKLM–induced MMP gene up-regulation in THP-1 cells at both mRNA and protein levels (Figure 3A and B). In contrast, TNF-alpha induction of MMP-9 was not affected in MyD88−/− cells as it activates MMP-9 gene expression via MyD88-independent pathway. Likewise, MyD-88 deficiency also completely decreased activation of NF-κB/AP-1 following HKLM treatment (Figure 3C). These data suggest that MyD88 has important role in the activation of NF-kappaB/AP-1 transcription factors for the induction of MMP-9 by HKLM.

### MAPK and NF-kappaB signaling pathways are involved in HKLM-induced MMP-9 upregulation

**Figure 4:**
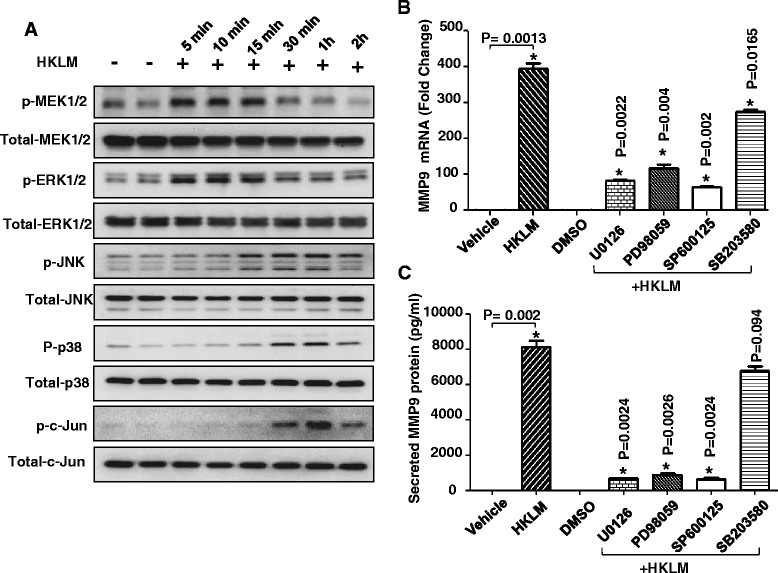
HKLM activates the MAP kinase signaling pathway. THP-1 cells were treated with HKLM for different time points and cell lysates were prepared as described in methods. Samples were run on denaturing gels. Phosphorylated MEK1/2, ERK1/2, JNK, p-38 and c-Jun are depicted in the upper panels and total respective proteins are shown in the lower panels. As shown, HKLM treatment increases the phosphorylation of MEK1/2, ERK1/2, JNK, p38 and c-Jun in a time-dependent manner **(A)**. THP-1 cells were pretreated with MEK-ERK inhibitors (U0126: 10 μM for 2 hr); PD98059: 10 μM for 1 hr) or JNK inhibitor (SP600125: 20 μM for 30 min) or p38 inhibitor (SB203580: 10 μM for 1 hr) and then treated with HKLM (3x10^7^/ml) for 24 hr. Cells and supernatants were collected. Cells were used for the isolation of total RNA and MMP-9 mRNA was assessed by real-time RT-PCR **(B)**. Secreted levels of MMP-9 protein were determined in supernatants by ELISA **(C)**. The results obtained from three independent experiments are shown. The data are presented as mean ± SE. An asterisk (*) represents *P*-value of <0.05.

**Figure 5:**
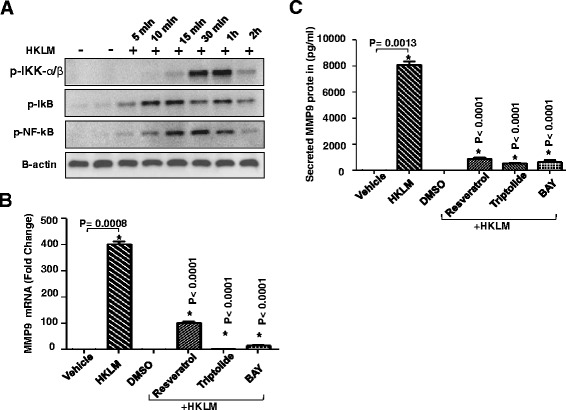
Effect of NF-kappaB pathway inhibitors on MMP-9 induction. THP-1 cells were treated with FSL-1 for different time points and cell lysates were prepared as described in methods. Samples were run on denaturing gels. Phosphorylated IKKα/β, IkB, and NF-κB are depicted in the upper panel and B-actin is shown in the bottom panel **(A)**. THP-1 cells were pretreated with NF-κB inhibitors (BAY 11–7085, 10 μM; Triptolide, 10 μM or Resveratrol, 15 uM) for 1 hr and then treated with HKLM (3x10^7^/ml) for 24 hr. Cells and supernatants were collected. Cells were used for the isolation of total RNA and MMP-9 mRNA was assessed by real-time PCR **(B)**. Secreted levels of MMP-9 protein were determined in supernatants by ELISA **(C)**. Data are shown as mean ± SE. An asterisk (*) represents *P*-value of <0.05.

Because activation of MEK/ERK and MAPK/JNK signalling pathways have been reported to mediate MMP-9 production [41,42], we next asked whether these molecules play role in HKLM induced MMP-9 production. Figure 4A showed that stimulation of THP-1 cells by HKLM increased phophorylation of MEK/ERK, JNK, p38, c-jun. As expected the Inhibitors of the MAPK pathway (MEK/ERK or p38 or JNK) down-regulate the expression of MMP-9 in THP-1 cells stimulated by HKLM. The expression of MMP-9 mRNA was reduced (Figure 4B; *P* < 0.05) by treatment with inhibitors of either MAPK/JNK (SB203580/SP600125) or MEK/ERK (PD98059, U0126). Consistent with qRT-PCR results, MMP-9 levels in culture supernatants of THP-1 cells were significantly suppressed (*P* < 0.05) after treatment with inhibitors of either MAPKs or MEK/ERK (Figure 4C). Given that the promoter contains the NF-κB binding site [43,44], the lack of NF-κB activation is expected to result in the reduced MMP-9 gene expression. Figure 5A showed that stimulation of THP-1 cells by HKLM increased phophorylation of IKK-α/β, p-IkB and NF-kappaB. The use of NF-κB inhibitors (BAY 11–7805, Triptolide and Resveratrol), significantly reduced the MMP-9 gene expression (*P* < 0.0001) (Figure 5B and C).

## Discussion

MMP-9 is essential for normal physiological conditions but its increase in production could be involved in the pathogenesis of various diseases such as chronic inflammation, tumor cell metastasis, arthritis, obesity and in the progression of various infectious diseases ([25-28]. Also, MMP involvement in the pathogenesis of central nervous system diseases have been reported. A study reported the presence of *Listeria monocytogenes* antigens and MMP-7/9 in sheep brains [45]. Previously, we studied the MMP-9 production induced by FSL-1, a synthetic lipoprotein derived from *Mycoplasma salivarium* [46]; whereas in this study, we have demonstrated that MMP-9 was up-regulated in monocytic cells upon exposure to Heat killed *Listeria monocytogenes* (HKLM). This MMP-9 up-regulation requires TLR2/MyD88-dependent activation of NF-κB/AP-1. Our study shows that HKLM mediated induction of MMP-9 in monocytic cells is due to HKLM interaction with surface receptor on the target cells. The interaction of different bacteria with TLRs has been shown to be decisive in the outcome of immune responses and bacterial pathogenesis. For instance, TLR2 serves as the signaling receptor for molecules such as LP, LTA, and PGN. PGN, a cell wall component of gram positive bacteria, induces IL-6 and MMP-9 gene expression in microglia and neurtophils, respectively 9 [47,48]. Other studies have also shown that macrophages from TLR2-deficient mice lost the ability to secrete the inflammatory cytokines TNF-alpha and IL-6 in response to bacterial component from gram-positive bacteria [49], which suggests that interaction of the TLR on the cells with the bacterial component is responsible, in part, to induce immune response in the host. In the present study, to demonstrate that TLR2 participates in the induction of MMP-9 by HKLM, we achieved no cellular responses to the HKLM after neutralization of TLR2 on THP-1 cells. Previous studies have shown that the cytokine production induced by gram positive bacteria in cells of the monocytic linage depends on TLR2 stimulation [50,51].

Recognition of a microbial invasion through the TLRs triggers the recruitment and activation of several adaptor proteins to the TIR domain. MyD88 is a key adaptor protein and is common to almost all TLRs except TLR3 [10]. The involvement of MyD88 is well known in the induction of various inflammatory mediators. Thus, we also suggested that inducing effect of HKLM on MMP-9 was blocked in MyD88 deficient cells. It is noteworthy in this regard that the role of MyD88 has provided a further evidence for the involvement of the TLR2 in this induction of MMP-9. The interaction of MyD88 with the TLR receptor promotes the recruitment of other adaptor proteins that in turn activates downstream kinases including NF-κB–inducing kinase (NIK), IKKα/β/γ, and mitogen-activated protein kinases (MAPKs) [13-18]. In the classical pathway, activated IKKβ which is part of an IKKα/β/γ complex, phosphorylates IKBα or IKBβ leading to their proteosomal degradation. As a result, NF-κB gets activated. In case of TLRs, che classical NF-κB pathway gets activated. Whereas in case of lymphoid development, the alternate pathway is involved in which NIK activates IKKα which phosphorylates NF-κB2 [52]. These signaling cascades thus activate multiple transcription factors such as NF-kappaB and activator protein 1 (AP-1) [53]. Indeed, previous studies indicate that AP-1 and NF-kappaB have binding sites in the MMP-9 promoter region and play important role in MMP-9 gene regulation [54]. Therefore, we show the involvement of NF-κB/AP-1 activation in HKLM-induced MMP-9 gene expression in THP-1 cells. This is also confirmed by our observations that neutralization of TLR2 and deficiency of MyD88 blocked NF-κB/AP-1 activation along with the inhibition of MMP-9 expression. As NF-kappaB and MAPK pathways have been extensively studied downstream of various inflammatory stimuli. It is well established that stimulation of monocytes/macrophages by microbial components induces phosphorylation of p38, ERK1/2, and c-Jun NH_2_-terminal kinase (JNK). MAPKs are activated largely by bacterial products through TLRs and participated in the inflammatory response induced by TLR2 activation in monocytes/macrophages [53,55]. Our results showed that HKLM induced phosphorylation of p38, ERK1/2, and c-Jun NH_2_-terminal kinase (JNK) are involved in the regulation of MMP-9 gene expression. Moreover, this is confirmed by our findings that HKLM induced MMP-9 was reduced by inhibition of MEK/ERK, JNK and p38. The involvement of MEK/ERK as well as that of other kinases (JNK and p38) in MMP-9 expression has been reported in other cell systems [54,56]. Since our data shows that NF-kappaB signaling pathways are also very effective in the regulation MMP-9 along with MAPK signaling pathways. Thus, we established that HKLM induced phosphorylation of the IKKα/β, IKB and NF-kappaB. Furthermore, we found that effect of HKLM on MMP-9 regulation was suppressed in the cells treated with inhibitors of NF-kappaB signaling pathways. Our results suggested that signaling pathways induced by HKLM that regulate MMP-9 expression in monocytic cells is also dependent on NF-kappaB signalling pathways. Many studies have revealed an essential role of NF-kappaB and AP-1 activation in MMP-9 secretion has been revealed in different cell types by several external stimuli [38,39]. A few studies support that NF-kappaB and AP-1 transcription factors are regulated by the similar intracellular signal transduction cascades [57-60]. For example, in the activation of JNK by inflammatory or stress stimuli and the nuclear traslocation of NF-kappaB, the simultaneous activation of NF-kappaB and AP-1 suggest that these transcription factors work cooperatively [61]. Another indication of the interaction between AP-1 and NF-kappaB activation pathways comes from the studies showing that the MAPK pathway activation leads to activation of JNK and IkB kinase complexes [62]. This cooperative interaction between AP-1 and NF-kappaB is further supported by the presence of a scaffold protein which was shown to be involved in the activation of JNK pathways and NF-kappaB nuclear translocation [63]. From these studies, taken together, there is possibility that NF-kappaB and AP-1 can modulate the activity of each other.

In summary, we have shown that TLR2 regulates the expression of MMP-9 in THP-1 cells in response to HKLM by multiple cooperative mechanisms. In particular, we have identified HKLM-mediated activation of MAPK, AP-1 and NF-κB signaling pathways as critical steps for transcriptional up-regulation of MMP-9.
